# Effects of Heat Stress-Induced Sex Hormone Dysregulation on Reproduction and Growth in Male Adolescents and Beneficial Foods

**DOI:** 10.3390/nu16173032

**Published:** 2024-09-08

**Authors:** Seong-Hee Ko

**Affiliations:** Major in Food Science and Nutrition, College of Human Ecology, Sookmyung Women’s University, Seoul 04310, Republic of Korea; marialovegod625@gmail.com; Tel.: +82-10-6533-2379

**Keywords:** testosterone, heat stress, male adolescents, delayed puberty, incomplete sexual maturation, reproductive failure, repairs of germ cell damage, foods, supplements

## Abstract

Heat stress due to climate warming can significantly affect the synthesis of sex hormones in male adolescents, which can impair the ability of the hypothalamus to secrete gonadotropin-releasing hormone on the hypothalamic–pituitary–gonadal axis, which leads to a decrease in luteinizing hormone and follicle-stimulating hormone, which ultimately negatively affects spermatogenesis and testosterone synthesis. For optimal spermatogenesis, the testicular temperature should be 2–6 °C lower than body temperature. Heat stress directly affects the testes, damaging them and reducing testosterone synthesis. Additionally, chronic heat stress abnormally increases the level of aromatase in Leydig cells, which increases estradiol synthesis while decreasing testosterone, leading to an imbalance of sex hormones and spermatogenesis failure. Low levels of testosterone in male adolescents lead to delayed puberty and incomplete sexual maturation, negatively affect height growth and bone mineral density, and can lead to a decrease in lean body mass and an increase in fat mass. In order for male adolescents to acquire healthy reproductive capacity, it is recommended to provide sufficient nutrition and energy, avoid exposure to heat stress, and provide foods and supplements to prevent or repair testosterone reduction, germ cell damage, and sperm count reduction caused by heat stress so that they can enter a healthy adulthood.

## 1. Introduction

In recent decades, extreme heat events have occurred frequently in many parts of the world, negatively impacting agriculture and a wide range of species. What is even more serious is the prospect that this abnormally high temperature phenomenon will gradually accelerate [1,2].

Prolonged exposure to high temperatures causes the body’s heat gain to exceed its ability to dissipate heat, preventing the body from properly regulating body temperature, resulting in an abnormal rise in core body temperature [3]. Environmental heat stress also affects the endocrine system and hormone synthesis, as well as leads to a decline in semen quality in human males, a major cause of male infertility [4,5]. However, accumulated research provides evidence that environmental heat stress has negative consequences for male reproduction [6,7]. Male reproductive function is more vulnerable to heat stress than female reproductive function due to several factors, mainly related to the sensitivity of the testes to temperature changes [8]. Germ cells such as spermatocytes are more susceptible to heat stress than other cells because of their high mitotic activity and lack of superoxide dismutase, and spermatocytes and early round spermatocytes are the most susceptible in humans [9,10]. In addition, heat stress mainly activates the mitochondrial apoptotic pathway in spermatocytes and sperm cells, affecting sperm integrity, causing DNA double-strand breaks, altering chromosome structure, and reducing chromatin material. Furthermore, during meiosis, heat stress causes abnormal segregation of sex chromosomes and the presence of an unpaired Y chromosome, which causes spermatocytes to undergo apoptosis [11]. In human and animal studies, heat stress has been proven to have negative effects on male reproductive function, including reducing testosterone and androgen levels [12], decreased sperm count [13], and causing male infertility [14].

Unfortunately, among the studies reported so far, there is no research on protecting male adolescent reproductive function from heat stress. Adolescence is the intermediate stage between childhood and adulthood and is the period when secondary sexual characteristics begin to appear and reproductive capacity is acquired [15]. Additionally, adolescence is a formative stage in life where patterns of growth, development, and behavior lay the foundation for health for later and future generations [16].

According to the World Health Organization (WHO), adolescence is defined as 10 to 19 years of age, and youth is defined as 15 to 24 years of age. Youth includes all age groups from 10 to 24 years old [15]. This study adopted a comprehensive age definition of 10 to 24 years and selected male adolescents as the subject of study. In 2019, the number of global adolescents aged 10 to 24 reached 1.8 billion, accounting for a quarter of the global population [17] and 90% of them live in low- and middle-income countries [18]. Analysis of the percentage of the total national population aged 10–24 years compared to the national population in 2019 shows that in most African countries, 30–35% of the total population belongs to this population group, which is easily exposed to environmental heat stress and is most susceptible to damage [15,18]. Considering that adolescent nutritional status can directly lead to adulthood health, protecting reproductive function in adolescence is an area that cannot be overlooked.

In this study, the effects of continuous heat stress on sexual hormonal synthesis, the hypothalamic–pituitary–gonadal (HPG) axis, and testicular function during male adolescence when secondary sexual characteristics are developing are discussed. Several studies have already verified that heat stress can interfere with sex hormone synthesis in male adolescents by causing oxidative stress, inflammation, and apoptosis within germ cells [12,13]. Based on these studies, the effects of dysregulated sex hormones in male adolescence on the reproductive system, height growth, bone mineral density (BMD), and body composition are discussed. Lastly, nutritional guidance and beneficial foods that help prevent or recover from decreased testosterone and infertility caused by heat stress in male adolescents are reviewed.

## 2. Effect of Heat Stress on Sexual Hormonal Synthesis in Male Adolescents

Heat stress due to climate warming can have significant effects on sex hormone synthesis in male adolescents. Here, a detailed overview of how heat stress can impact the endocrine system and hormone synthesis is provided.

### 2.1. Impact on the Hypothalamic–Pituitary–Gonadal (HPG) Axis

**Figure 1:**
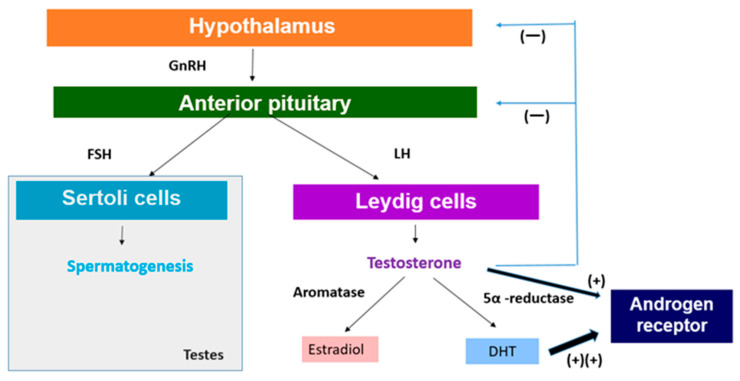
Regulation of sex hormone production. GnRH: gonadotropin-releasing hormone, LH: luteinizing hormone, FSH: follicle-stimulating hormone, DHT: 5α-dihydrotestosterone.

The HPG axis is crucial for the regulation of sex hormone production. Heat stress can disrupt this axis in several ways [19]. With the onset of puberty, the hypothalamic–pituitary–gonadal axis begins to regulate gonadotropin levels and gonadal function [20]. The outline is briefly as follows (Figure 1). The hypothalamus releases several tropic hormones along with gonadotropin-releasing hormone (GnRH), a peptide hormone that controls the HPG axis. GnRH is released into the hypothalamic–pituitary portal system, which consists of two connected capillary beds that transport the hormone directly from the hypothalamus to the pituitary gland [21]. The pituitary gland is composed of the anterior lobe and the posterior lobe. The anterior pituitary is associated with the HPG axis, and GnRH stimulation of gonadotropin cells results in the release of luteinizing hormone (LH) and follicle-stimulating hormone (FSH) [22].

In men, FSH acts on Sertoli cells in the testes to stimulate spermatogenesis (Figure 1) [22]. LH acts on interstitial Leydig cells to stimulate the production and release of testosterone (Figure 1) [23]. GnRH production increases significantly at the onset of puberty, which leads to the release of gonadotropins that stimulate spermatogenesis and testosterone production [20]. On the other hand, the secretion of anterior pituitary hormones is regulated through hormone release and inhibition in the hypothalamus [20]. For growth hormone (GH), it secretes growth hormone-releasing hormone (GHRH) to stimulate its release and somatostatin to inhibit it. For LH and FSH, the hypothalamus releases GnRH in a pulsatile manner to stimulate their secretion [24]. Testosterone produced by interstitial Leydig cells undergoes negative feedback to maintain appropriate testosterone levels in the body [25]. Testosterone functions to feedback and inhibit the release of GnRH from the hypothalamus as well as to suppress the release of gonadotropins by the anterior pituitary (Figure 1) [26].

**Figure 2:**
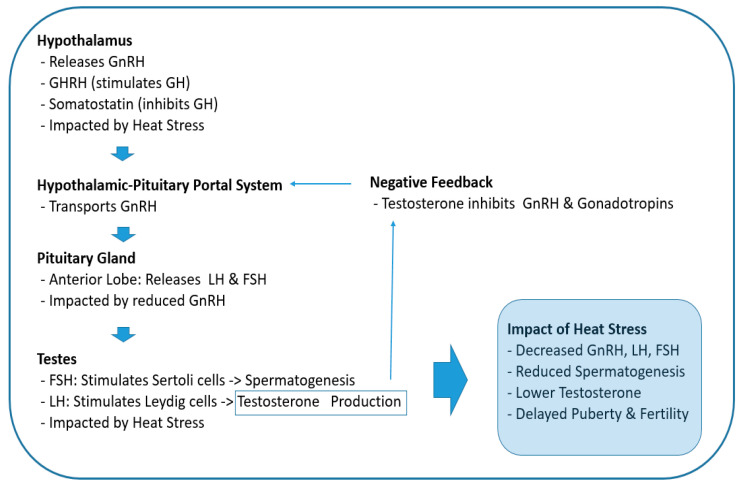
Effects of heat stress on the hypothalamic–pituitary–gonadal axis during puberty. GnRH: gonadotropin-releasing hormone, GH: growth hormone, GHRH: growth hormone-releasing hormone, LH: luteinizing hormone, FSH: follicle-stimulating hormone.

However, heat stress can impair the hypothalamus’s ability to secrete GnRH, which is crucial for stimulating the pituitary gland to release LH and FSH (Figure 2). This disruption can negatively impact reproductive functions in mammals [27]. For instance, research on Wenchang chicks demonstrated that heat stress alters the expression of GnRH in the hypothalamus, which subsequently affects the levels of LH and FSH, leading to reproductive dysfunctions [28]. Similarly, studies in dairy cows have revealed that heat stress reduces the expression of hypothalamic kisspeptin, an upstream regulator of the HPG axis, thereby impacting the release of GnRH and consequently lowering the levels of LH and FSH [29,30].

Kisspeptin is a ligand for the G-protein-coupled receptor GPR54 (also known as Kiss1R) and is encoded by the *KISS1* gene in humans, which is an essential upstream regulator of GnRH secretion, the major hypothalamic node for the stimulation of the HPG axis [31]. In other words, kisspeptin is essential for stimulating GnRH release, so a decrease in the expression of kisspeptin reduces GnRH release in the hypothalamus. Further, investigations in cattle have found that heat stress disrupts the hormonal balance necessary for successful reproduction. Its decrease compromises sperm quality, which leads to lower fertility rates [32].

In the pituitary gland, the production of LH and FSH decreases due to reduced GnRH secretion. Likewise, as gonadotropin (LH and FSH) levels decrease, sex steroid levels also decrease (Figure 2). In testes, FSH acts on Sertoli cells to stimulate spermatogenesis, and LH acts on the smooth endoplasmic reticulum of Leydig cells to stimulate the production and release of testosterone (Figure 2) [33]. Therefore, reduced levels of these hormones due to heat stress at the onset of puberty ultimately has a negative impact on spermatogenesis and testosterone synthesis, which may lead to delayed puberty and fertility issues [34].

### 2.2. Direct Effects on Testicular Function

The testes are particularly sensitive to temperature changes. Elevated temperatures can lead to impaired spermatogenesis, reduced testosterone synthesis, and Leydig cell dysfunction [35].

Mammalian spermatogenesis is a temperature-sensitive process, and increases in testicular temperature impair spermatogenesis. For optimal spermatogenesis under normal conditions, testicular temperature must be 2 to 6 °C lower than body temperature [36]. The spermatogenic process proceeds with blood and oxygen supply fairly independent of other vascular bed changes in the body [37]. Despite this well-controlled local environment, testicular temperature may be elevated by heat stress, and higher temperatures may result in increased testicular metabolism without increased blood supply, leading to local hypoxia and detrimental effects on the tissue [37,38]. That is, elevated testicular temperature can cause hypoxia-reperfusion injury, which can lead to oxidative imbalance after normothermia is restored and tissues are reperfused [37], which is similar to the hypoxia-reperfusion injury that occurs during organ transplantation [39].

Heat stress has been shown to reduce testosterone synthesis by damaging the testicles, which is due to various mechanisms, such as increased oxidative stress, disruption of steroidogenic enzyme expression, and apoptosis of Leydig cells [12]. Moreover, heat stress causes oxidative stress to the testicles, which increases the production of reactive oxygen species (ROS) and lipid peroxidation while decreasing antioxidant defenses [40]. This oxidation environment can damage Leydig cells, which can damage testosterone generation. Particularly, heat stress reduces the expression of major steroids such as 3β-HSD (3β-Hydroxysteroid Dehydrogenase), CYP11A1(Cytochrome P450 Family 11 Subfamily A Member 1), HSD3B1(Hydroxysteroid 3β-Dehydrogenase Type 1), and Cytochrome P450 series (CYP17A1), which are essential for the biosynthesis of testosterone [41]. In addition to this, heat stress causes histopathological changes in Leydig cells, such as dilated smooth endoplasmic reticulum and enlarged mitochondria, which further contribute to their dysfunction [42].

Recently, various animal and human studies have been reported on the adverse effects of heat stress on male reproductive function. In animal experiments, various studies have been reported, including studies on the modification of sheep sperm surface proteins by heat stress [43], studies on ruminants showing reduced fertility [44], and studies on the reduction of testosterone synthesis using rodents [45,46]. In human studies, it has been demonstrated that varicose veins reduce human fertility [47] and that male germ cells are sensitive to heat stress due to testicular heat stress [48,49]. In a recent epidemiological study, a population-based longitudinal study involving 10,802 Chinese men reported that when air temperature exposure was ≥13 °C, each 5 °C higher temperature was significantly associated with a 0.70 × 10^6^/^mL^, 4.09 × 10^6^, 1.01%, 1.06%, 4.31 × 10^6^, and 4.20 × 10^6^ decrease in sperm concentration, total sperm count, total motility, progressive motility, total motile sperm count and progressively motile sperm count, respectively [50]. Additionally, a multicenter retrospective cohort study was conducted to investigate the exposure-response relationship between temperature anomalies (TA) that deviate from long-term climate patterns and sperm quality. A total of 78,952 sperm samples from 33,234 donors from six provinces in China were analyzed, and heat-related TA in the hot season was significantly negatively associated with sperm concentration, progressive sperm count, and total motile sperm count (all *p* values < 0.05) [51].

The overall results show that heat stress significantly reduces serum and testicular testosterone levels, negatively affects total sperm count, sperm motility, and sperm quality, and may lead to male fertility issues.

### 2.3. Chronic Heat Stress Can Lead to an Imbalance of Testosterone and Estradiol

Spermatogenesis requires an appropriate balance between testicular testosterone and estradiol [26]. Testosterone is produced by Leydig cells, which are involved in testicular steroidogenesis, and is converted to estradiol by cytochrome P450 aromatase [52]. Estradiol is involved in male sexual differentiation and spermatogenesis, and affects sperm motility, receptivity, and survival [26]. Aromatase is expressed in Leydig cells, Sertoli cells, and germ cells of the mammalian testis, and Leydig cells have been considered the major source of estradiol [52]. Therefore, regulation of aromatase expression in Leydig cells is essential for regulating the endocrine environment between the testes.

Recently, Oka et al. reported that chronic heat stress (40 °C for over 12 h) stimulated aromatase transcription in R2C rat Leydig tumor cells, whereas acute heat stress did not affect transcription [53], which suggests that the increase in aromatase levels in Leydig cells due to chronic heat stress is a factor causing an imbalance between testosterone and estradiol in men with spermatogenesis failure. For example, the seminal plasma and serum of infertile men have been reported to have high estradiol levels [54] and low testosterone to estradiol ratios [55]. In addition, it has been evaluated that the quality of semen of workers exposed to heat stress in the steel industry is significantly reduced [56]. Based on these findings, avoiding exposure to chronic heat stress may be necessary to achieve a balanced testosterone to estradiol ratio in male adolescence. Based on these findings, avoidance of exposure to chronic heat stress is necessary to maintain a balanced testosterone-to-estradiol ratio and protect reproductive function in male adolescence.

## 3. Effects of Dysregulated Sexual Hormone during Adolescence on Reproductive System and Growth

It is well known that testosterone exerts its effect by binding to androgen receptor (AR), a ligand-dependent nuclear transcription factor. AR is expressed in various, such as reproductive, skeleton, muscle, adipose tissue, cardiovascular, neurological, and hematopoietic systems [26]. Therefore, the expression of testosterone affects not only reproduction but also various organs. In this chapter, we will explore the effects of the decrease in testosterone in relation to the reproduction and growth of adolescents (Figure 1).

### 3.1. Low Levels of Testosterone and Reproductive System

It has been drawing attention that the dysfunction of HPG axis caused by heat stress can ultimately lead to decreased testosterone levels [27,57]. As already mentioned, GnRH production increases significantly at the onset of puberty, which leads to the release of gonadotropins that stimulate spermatogenesis and testosterone production [15]. If the level of GnRH released from the hypothalamus is reduced due to heat stress, the secretion of LH and FSH produced by GnRH stimulating the pituitary can also be reduced [30]. As a result, the production of testosterone, which is produced by stimulating Leydig cells in the testis, is also reduced [58].

In order to understand the possible impact of decreased testosterone levels on the development of secondary sexual characteristics during puberty, it is necessary to look at the metabolism of testosterone. Testosterone is metabolized by two major enzyme aromatases and 5α-reductase (Figure 1) [52]. Aromatases are expressed in bone, breast, brain, and adipose tissue as well as male gonads and catalyze the binding of testosterone to estradiol [52]. It is also expressed in tissues and catalyzes the conversion of testosterone to estradiol by binding to estrogen receptors [59]. Estradiol, commonly recognized as a female sex hormone, also plays significant roles in male reproductive health, including promoting sperm production, sexual desire, and erectile function [60]. Testosterone is converted to 5α-dihydrotestosterone (DHT) by the enzyme 5α-reductase, which plays a significant role in male sexual differentiation and development [61], which is mainly expressed in the prostate, liver, and skin (Figure 1) [62]. DHT is the most potent androgen and binds to AR with the highest affinity and is primarily responsible for facial and body hair growth in addition to prostate growth at the onset of puberty (Figure 1) [63]. Therefore, a decrease in testosterone levels will also lead to a decrease in the levels of DHT, which is responsible for adolescent prostate growth and facial and body hair, which may negative affect adolescent secondary sexual characteristics.

Ultimately, the effects of decreased testosterone during adolescence on the reproductive system are primarily characterized by delayed or incomplete puberty. Delayed puberty is characterized by a lack of secondary sexual characteristics such as deepening of the voice, growth of facial and body hair, and increased muscle mass [64]. Incomplete puberty means partial development of these characteristics [65]. The crucial role of testosterone is in the initiation and progression of puberty, which stimulates the growth and development of the male reproductive system and secondary sexual characteristics [20]. Therefore, low testosterone levels can delay the start of this process, delaying puberty.

Testosterone also plays an absolutely important role in testicular size. It is essential for testicular growth and maturation because it supports sperm production by stimulating Sertoli cells within the testes [66]. In the absence of an appropriate level of testosterone, the growth of the testis slows, resulting in smaller testes, which can also be confirmed in the circulating testosterone levels of patients with hypogonadism [67]. Circulating testosterone levels in patients with congenital hypogonadotropic hypogonadism are typically low, <3 nmol/L (86.5 ng/dL) [68]. In a recent retrospective chart analysis study targeting 24,440 Vietnamese adult men, it was reported that testicular size and testosterone are positively correlated [69]. Additionally, testicular size was unrelated to the subject’s age and smoking habits, and it was reported that men with hypogonadism and unexplained infertility had significantly smaller testes than healthy men [69].

Moreover, testosterone plays an important role in spermatogenesis, which is necessary to maintain the development of sperm cells and the health of the seminiferous tubules, where spermatogenesis occurs. Low testosterone levels can reduce sperm production and function, leading to symptoms such as low sperm count and azoospermia, which can ultimately affect fertility [62,67].

### 3.2. Low Levels of Testosterone Impact on Height and Bone Mineral Density in Adolescence

Testosterone plays a crucial role in promoting bone growth and density during adolescence. It is already well known that BMD in adolescence peaks in the late teens and early 20s [70]. In addition, in the two years of peak skeletal growth, adolescents accumulate more than 25 percent of their adult bone mass, and the bone density that forms at this time becomes a major determinant of osteoporosis and its complications in adulthood [71]. Decreased testosterone synthesis can lead to lower BMD and potentially increase the risk of osteoporosis in elderly life, which is because testosterone is an important hormone for bone formation and maintenance [26].

Generally, adolescence is characterized by rapid bone growth. It has been reported that growth hormone acts as a major factor in rapidly increasing epiphyseal growth during adolescence [72]. Testosterone and other androgens act to increase longitudinal bone growth and have been shown to act through estrogen receptors [73]. As a result, it was proven that the higher the testosterone level in humans, the greater the height [74], which shows that testosterone plays an important role in height growth in male adolescence. As mentioned earlier, testosterone binds to estrogen receptors and is converted to estradiol by aromatase. Heat stress can impair the function of the tests, which are responsible for producing testosterone [32]. Thus, reduced testosterone synthesis subsequently leads to a decrease in the amount of testosterone available for conversion into estradiol [75]. Therefore, a decrease in testosterone during adolescence, when rapid bone growth occurs, may have a negative effect on height growth.

Until now, many studies have reported that testosterone is involved in bone density in men. As men age, testosterone levels naturally decline and BMD decreases [76,77]. However, it was demonstrated that testosterone treatment in elderly men with hypogonadism increases BMD [78]. Additionally, Adrian et al. demonstrated that physiologic testosterone treatment increases BMD at the hips while maintaining BMD at the spine in female-to-male transgender patients, which suggest that this may be the result of a direct action of the testosterone hormone on bone or an indirect action through aromatization to estradiol [79]. These kinds of studies show that a decrease in testosterone can negatively affect height growth and BMD in adolescence.

### 3.3. Low Testosterone Influence Body Composition in Adolescence

Changes in body composition during adolescence are distinctly different depending on gender. Before puberty, the ratio of lean body mass (LBM) and fat mass (FM) was similar between men and women, with body fat being about 16–18% and muscle strength being roughly the same [80]. During adolescence, women’s fat tissue increases relatively more than men’s, and for men, lean tissue (muscle and bone) increases significantly. Until adulthood, women have more than twice as much body fat as men, while men have the characteristic of having 50% more lean tissue than women [81]. The difference in body fat between genders in normal adults (23% for women and 12% for men) occurs during puberty. Therefore, the body composition formed during puberty plays a role in determining sexual characteristics in adulthood [82]. Therefore, the body composition formed during puberty plays a role in determining sexual characteristics in adulthood. During this period of sexual development, testosterone has a significant impact on body composition through its interactions within fat and muscle tissue [26].

Excitingly, Santosa et al. demonstrated that testosterone inhibits fatty acid storage through inhibition of lipoprotein lipase (LPL) and acyl coenzyme A synthetase (ACS) activities [83]. LPL catalyzes the conversion of triacylglycerol(TG) into free fatty acids (FFAs) and glycerol for uptake and storage of TG by the adipocytes and plays a major role in distribution of fat stores [59]. On the other hand, FFA is mainly used for energy storage in adipose tissue [59]. ACS is necessary for the transportation of fatty acids (FAs) into cells, which leads to initiate the activation of those FAs through catalyzing the formation of fatty acyl CoA [84]. Moreover, Bhasin et al. reported that testosterone stimulates lipolysis by inhibiting lipid absorption and the activity of low-density lipoprotein and increasing the number of β-adrenergic receptors [85]. Actually, β3-adrenoceptors are traditionally recognized for their role in adipose tissue where they mediate lipolysis. Activation of these receptors stimulates the release of fatty acids from triglycerides stored in adipocytes, which are then used for energy production through β-oxidation in mitochondria [86]. In other words, testosterone reduces the activity of LPL and ACS, inhibits lipogenesis and accumulation of fat tissue, and stimulates lipolysis.

As already explained, since AR is expressed in skeletal muscle, testosterone, which binds to it and exerts its effects, also affects skeletal muscle [26]. Griggs et al. reported that testosterone increases muscle protein synthesis (MPS) without affecting whole-body protein synthesis [87], which is owing to the fact that testosterone increases the expression of insulin like growth factor 1 (IGF1) in muscle [88]. It has also been shown to increase anabolic pathways and hypertrophy when administered to skeletal muscle [89]. Additionally, Shefeld-Moore et al. reported that when oxandrolone, a synthetic analogue of testosterone, was orally administered to young men [22 ± 1 (±SE) year], the expression of AR increased in skeletal muscle samples and at the same time, intracellular reutilization of amino acids was improved, thereby increasing anabolism [90]. Clinically, testosterone administration has been shown to increase lean body mass and maximal voluntary muscle strength in a dose-dependent manner [85], which can be explained by the finding that androgens interact with AR to promote the myogenic differentiation and inhibit adipogenesis [91].

The evidence has consistently been reported that a decrease in serum testosterone is related to a decrease in LBM [92]. Katznelson et al. have previously reported in a study of healthy, hypogonadal men with low serum testosterone levels that they had lower LBM and higher fat mass compared to age-matched eugonadal men [93]. Furthermore, it was examined that experimental suppression of serum testosterone by administering the GnRH agonist analogue to healthy young men was associated with a significant decrease in LBM, an increase in fat mass, and a decrease in muscle mass [94,95]. Based on these findings, considering the characteristics of body composition in adolescence, it can be seen that a decrease in testosterone leads to a decrease in LBM and an increase in FM.

## 4. Beneficial Foods That Help Prevent or Recover from Low Testosterone and Infertility Caused by Heat Stress

Up to now, heat stress has been reported to have negative effects on male reproductive hormones and germ cells, including a decrease in testosterone. When testosterone levels are low, topical or injected testosterone can decrease sperm production through negative feedback to the hypothalamus and pituitary gland, which can lead to the risk of infertility [96]. In order to actively cope with this inevitable trend, this chapter examines the energy required for adolescent reproductive growth, and foods and supplements that help with testosterone reduction, sperm count protection, and sperm repair due to heat stress.

### 4.1. Metabolism and Energy Requirement for Male Adolescence

Since adolescence is a period of rapid growth, adequate nutrition is very important to achieve full growth potential, and failure to achieve optimal nutrition can lead to growth retardation and stunting. The timing and duration of body composition changes are directly linked to sexual maturation. Therefore, nutritional requirements depend more on sexual maturity than chronological age [97].

Metabolism is directly related to total energy requirements and indirectly to growth, which consists of energy cost of growth (ECG), basal metabolic rate (BMR), and activity energy expenditure (AEE). BMR is the energy expended by internal processes during complete rest in a climate-controlled environment for at least 10 to 12 h after consuming the most recent meal. That is, it is the minimum amount of energy required to sustain life processes [98], which increases rapidly until age 2 and declines throughout adolescence [99]. However, if total energy intake falls below BMR, ECG and AEE may be impaired, leading to growth failure, delayed puberty, and impaired bone mass accumulation in male adolescence [97]. Therefore, adequate total energy intake is essential to initiate normal puberty. According to WHO, the Schofield equation is used to estimate BMR considering gender, age, and weight [100]. For males 10–18 years of age, BMR (megajoules (MJ)/day) = (0.074 × body weight (kg)) + 2.754 and BMR (kcal/day) = (17.69 × body weight (kg)) + 658.

### 4.2. The Role of Melatonin in Preventing Reproductive Damage Caused by Heat Stress

Melatonin (N-acetyl-5-methoxytryptamine) is widely distributed and has a wide range of functions that are well known to regulate cellular physiology and molecular biology [101]. Many of these actions are mediated by well-characterized G-protein-coupled melatonin receptors in the cell membrane, and its ability to detoxify reactive oxygen species and related oxygen derivatives allows it to affect cellular molecular physiology through receptor-independent means [102]. Melatonin has many potential applications in both human and veterinary medicine because it readily crosses physiological barriers, such as the blood–testis barrier, and is virtually nontoxic [101]. Among its many physiological regulatory functions, one that deserves our attention is its ability to reduce oxidative stress induced by heat stress and repair the damage it causes.

A recent study using boar Sertoli cells demonstrated that melatonin alleviates the heat stress-induced impairment of Sertoli cells by reprogramming glucose metabolism when melatonin was pretreated for 1 h or 3 min 24 h before heat stress [103]. Sertoli cells, the only somatic cells in direct contact with germ cells, provide structural and nutritional support for developing germ cells, and normal glucose metabolism is essential for spermatogenesis [104]. Melatonin can exert its antioxidant effects by directly scavenging ROS or by activating antioxidant defense systems [101]. On the other hand, the concentration range of melatonin in porcine seminal plasma is 2.75–35.61 pg/mL [105], and melatonin membrane receptors have been found in mouse, pig, and human testis, which suggests that melatonin binds to melatonin membrane receptors and participates in the regulation of spermatogenesis [105,106].

Dang et al. have shown that heat stress leads to oxidative stress, damaged mitochondria, and the pentose phosphate pathway, which in turn leads to Sertoli cell apoptosis [103]. In addition, it was verified that melatonin exerted antioxidant effects through the NRF2/KEAP1 antioxidant pathway and successfully rescued heat stress-induced damage by reprogramming the glucose metabolism of Sertoli cells through the MTNR1B–HSP90–HIF-1α axis [103]. Melatonin increased the expression of heat shock protein 90 (HSP 90) through the melatonin receptor 1B (MTNR1B), which stabilizes hypoxia-inducible factor-1α (HIF-1α) [103]. Activation of the HIF-1α signaling pathway increased glycolysis, promoted the pentose phosphate pathway, and increased cell viability [103]. These results provide a theoretical basis for melatonin to regulate cellular energy metabolism and oxidative stress, which are essential for preventing and eliminating reproductive disorders caused by heat stress.

In an animal model studying the reproductive function of male dairy cows, it was found that melatonin treatment could upregulate the gene expression of MT2 (Metallothionein-2A), which was downregulated by heat stress, improve the changes in the extracellular matrix components, and restore the serum testosterone level [107]. For the first time, it was examined that melatonin could prevent testicular and sperm cell damage and improve semen quality in heat-stressed male dairy goats by inhibiting the PI3K/AKT signalling pathway [107]. Ultimately, this study provided the basis for the molecular mechanism of melatonin to protect the male reproductive process under heat stress and to use exogenous melatonin to prevent heat stress.

Importantly, the reproductive protective effect of melatonin against heat stress using human spermatozoa was reported. Zhao et al. first demonstrated that heat stress-induced oxidative stress damages human sperm by decreasing sperm motility and viability [108]. It was also demonstrated that pre-treatment of human sperm with melatonin could alleviate this damage by inhibiting sperm mitochondrial ROS production, increasing mitochondrial membrane potential, reducing the formation of lipid peroxidation product 4-HNE (4-hydroxy-2-nonenal), and reducing sperm DNA damage and apoptosis [108]. These findings suggest that melatonin may be used as a preventive agent for male reproductive function damage caused by heat-induced oxidative stress.

### 4.3. Supplements to Repair Reproductive Damage Caused by Heat Stress

#### 4.3.1. Tanshinone IIA

Tanshinone IIA (TSA) is a natural diterpene quinone and a lipophilic component obtained from the root extract of Salvia miltiorrhiza [109]. Tanshinone IIA has antioxidant and anti-apoptotic effects and has been reported as a protective agent against heat shock [110]. Bai et al. verified that heat stress significantly decreased the diameter of the seminiferous tubules in mice, increased apoptosis in testicular tissue, and significantly reduced testosterone levels [46]. On the other hand, in the group that received Tanshinone IIA and heat stress simultaneously, the testicular morphology and apoptosis were significantly improved, and the testosterone secretion function was verified to be restored. Western blot technology demonstrated that the group receiving heat stress upregulated the expression of TGFβ1/Smad2/Smad3 pathway proteins, which induced apoptosis, testicular tissue organic lesions, and affected testicular secretory function. In addition, it was verified for the first time that Tanshinone IIA intervention could recover testicular damage induced by heat stress by inhibiting the expression of TGFβ1/Smad2/Smad3 pathway proteins [46]. This study verified that Tanshinone IIA is a supplement that can effectively recover testicular damage induced by heat stress in mice by inhibiting the TGFβ1/Smad2/Smad3 pathway and suppressing apoptosis.

#### 4.3.2. Melatonin

In vivo studies using mice have demonstrated that long-term melatonin supplementation can accelerate testicular recovery from heat stress-induced germ cell loss and tissue morphology disruption, which is based on the function of melatonin to promote the removal of apoptotic germ cells through RAC1-mediated phagocytosis by Sertoli cells, and to support germ cell regeneration by restoring tight and gap junctions [5]. These findings suggest that melatonin is a potential treatment for heat stress-induced sperm quality decline and male infertility.

Importantly, human studies using melatonin have also been reported. Long-term (45 days) daily oral supplementation of 6 mg of melatonin in infertile men increased endogenous melatonin levels, as indirectly measured by urinary 6-sulfatoxymelatonin (aMT6-s), improved total antioxidant capacity of both urine and seminal fluid, and consequently, reduced oxidative damage to sperm DNA [111].

Melatonin can be consumed through melatonin-rich foods in the diet; animal foods that are high in melatonin include eggs and fish, and among plant foods, nuts contain the highest levels of melatonin. Some types of mushrooms, grains, sprouted legumes, or seeds are also good dietary sources of melatonin [112]. In addition, it has been demonstrated that consumption of tropical fruits such as oranges, pineapples, and bananas increases serum melatonin concentrations and increases antioxidant capacity in the serum of healthy men in proportion to serum melatonin levels [107,113]. In addition, studies have reported that the Mediterranean diet contains a lot of plant foods rich in biologically active phytochemicals such as melatonin [114].

#### 4.3.3. *Lycium barbarum* Polysaccharide

*Lycium barbarum* polysaccharides (LBP), the major physiologically active components of *Lycium barbarum*, have been demonstrated to reverse heat stress-induced Sertoli cell dedifferentiation and improve the structural integrity of the blood–testis barrier. In addition, it was demonstrated that LBP improved heat stress-induced damage to primary Sertoli cells in rats by increasing the expression of androgen receptors and activating the Akt signaling pathway [115].

#### 4.3.4. Zinc Sulfate and Folic Acid

Combined dietary supplementation of zinc sulfate and folic acid under heat stress conditions improved testicular hemodynamics, testicular volume, plasma testosterone levels, and overall semen quality in rams [116].

#### 4.3.5. Flavonoids

Recently, it has been reported that foods rich in flavonoids can protect the testis from damage caused by heavy metals such as cadmium [117]. In a murine model, after CdCl_2_ was administered, molecular biological experiments were performed in the testis, and the results showed that it increased the mRNA of IL-1β, TNF-α, p53, and BAX, while decreasing the mRNA of Bcl-2, and induced tubular lesions and apoptosis of germ cells. On the other hand, when CdC_2_ lwas administered together with bergamot juice, curcumin, and resveratrol, it had a positive effect on Cd-induced testicular damage, suggesting that it can protect the testicular function of subjects exposed to environmental toxicants.

## 5. Conclusions

Clinical research and experiments on testosterone and estradiol imbalance and germ cell damage caused by heat stress in male adolescents are necessary areas for entering healthy adulthood. It is necessary to actively cope with changing warming trends to prevent puberty delays or incomplete sexual maturity due to heat stress, and to protect the reproductive rights of adolescents in order to form a healthy human future. It is important to note that it is necessary to provide sufficient nutrition and energy to ensure that adolescents’ sexual maturity occurs, to avoid exposure to heat stress as much as possible, and to provide food and supplements to prevent or repair testosterone reduction, germ cell damage, and sperm count reduction caused by heat stress. The strength of this review is that it comprehensively summarizes the changes in sex hormone synthesis that can occur when exposed to heat stress in male adolescents who are developing secondary sexual characteristics, and the resulting effects on reproductive dysfunction and height, BMD, and body composition in male adolescents with low testosterone, and also reviewed supplements that can help prevent or repair them. On the other hand, there is a limitation that the review was written based on adult reproductive studies and animal model studies, as there is currently no study conducted on the effects of heat stress on sex hormone regulation and reproduction in male adolescence. Supplements to prevent reproductive damage caused by heat stress include melatonin, and supplements to repair it include Tanshinone IIA, melatonin, *Lycium barbarum* Polysaccharide, zinc sulfate, and folic acid, but more research is needed.
